# Frontal Fibrosing Alopecia Mimicking Alopecia Syphilitica

**DOI:** 10.7759/cureus.21901

**Published:** 2022-02-04

**Authors:** Kyra L Diehl, Christof P Erickson, Antoanella Calame, Philip R Cohen

**Affiliations:** 1 Osteopathic Medicine, Western University of Health Sciences, Pomona, USA; 2 Dermatology, Compass Dermatopathology, San Diego, USA; 3 Dermatology/Dermatopathology, Compass Dermatopathology, San Diego, USA; 4 Dermatology, Scripps Memorial Hospital, La Jolla, USA; 5 Dermatology, University of California, Davis Medical Center, Sacramento, USA

**Keywords:** alopecia, androgenetic, areata, hair, fibrosing, frontal, mimicking, scalp, scarring, syphilis

## Abstract

Frontal fibrosing alopecia is lymphocytic scarring alopecia most commonly affecting postmenopausal women. Alopecia syphilitica, an uncommon manifestation of secondary syphilis, is characterized as a nonscarring and non-inflammatory hair loss that primarily affects the scalp. Frontal fibrosing alopecia has a classic pattern of hair loss involving regression of frontotemporal hair; it also may affect the eyebrows or other sites of the body. The typical patterns of frontal fibrosing alopecia are characterized as diffuse and linear. In addition, patients with frontal fibrosing alopecia can have atypical signs and patterns of hair loss. The atypical signs and patterns of frontal fibrosing alopecia are the androgenetic-like pattern, clown alopecia pattern, cockade-like pattern, doll hairline sign, lonely hair sign, ophiasis-like pattern, pseudo-fringe sign, and upsilon pattern. We observed a woman with a traditional pattern of frontal fibrosing alopecia whose hair loss involved the frontotemporal scalp areas; however, she also had hair loss in the occipital scalp that appeared similar to the moth-eaten alopecia of alopecia syphilitica. Her rapid plasma reagin was negative and the biopsies from her frontal scalp and occipital scalp both showed scarring alopecia consistent with frontal fibrosing alopecia. Her alopecia persisted with conservative treatment, and she returned to wearing a wig. Alopecia syphilitica-like pattern of hair loss can be added to the other atypical patterns of alopecia that may potentially be observed in a patient with frontal fibrosing alopecia.

## Introduction

Frontal fibrosing alopecia is a scarring alopecia. It classically presents with frontotemporal hair loss in postmenopausal women. However, atypical patterns of hair loss in patients with frontal fibrosing alopecia have been observed [1-3]. 

Alopecia syphilitica is nonscarring alopecia. It can be a manifestation of secondary syphilis. It can occur with other cutaneous features of syphilis or by itself [4].

A postmenopausal woman presented for evaluation of not only progressive regression of her frontotemporal hairline of several years duration but also patchy alopecia of her occipital scalp. Clinically, her hair loss was suggestive of frontal fibrosing alopecia and alopecia syphilitica. However, her rapid plasma reagin was negative and multiple scalp biopsies were consistent with frontal fibrosing alopecia. This paper emphasizes that frontal fibrosing alopecia can mimic alopecia syphilitica. Also, in addition to alopecia syphilitica-like alopecia, other atypical presentations of hair loss associated with frontal fibrosing alopecia are reviewed.

## Case presentation

**Figure 1:**
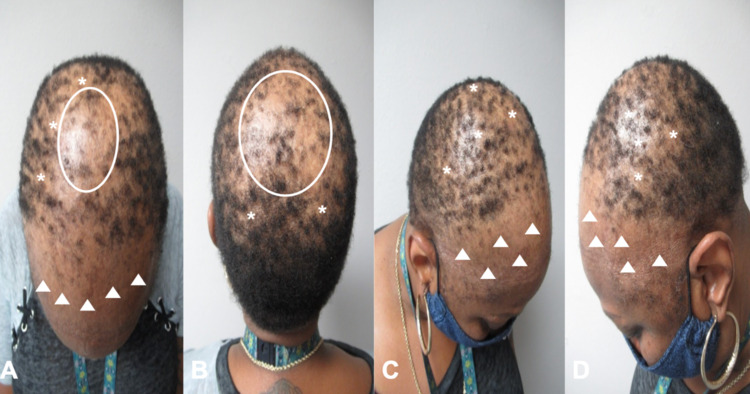
Clinical presentation of frontal fibrosing alopecia Anterior (A), posterior (B), right side (C), and left side (D) views of progressive frontotemporal hair loss (white triangles) and a receding hairline. There are also large areas of alopecia (white ovals) on the crown of the scalp with areas of patchy, moth-eaten alopecia (white asterisks) mimicking alopecia syphilitica.

**Figure 2:**
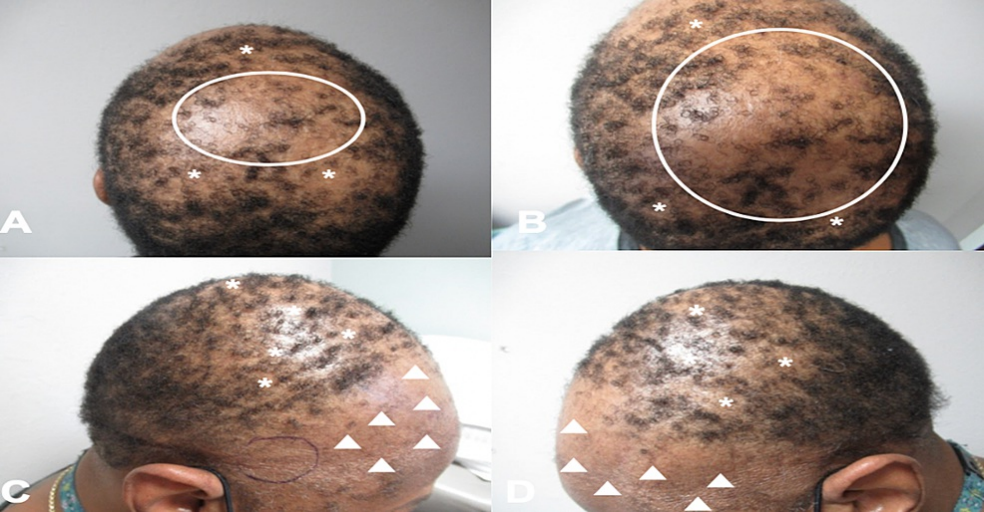
Frontal fibrosing alopecia presenting with mixed pattern of hair loss Distant (A) and closer (B-D) views of the posterior scalp (A, B), right anterior scalp (C), and left anterior scalp (D) show large areas of alopecia (white ovals) with areas of patchy, moth-eaten alopecia (white asterisks) mimicking alopecia syphilitica and progressive frontal hair loss with retraction of the hairline (white triangles). The purple oval on the right temple (C) is the planned site for monthly intralesional triamcinolone injections; a similar location on the left temple would also receive monthly injections.

An asymptomatic 56-year-old postmenopausal woman presented with progressive hair loss of more than three years duration. Cutaneous examination showed diffuse, frontotemporal hair loss with a receding hairline; there was also diffuse hair loss on the crown of her scalp (Figures 1A-1D, 2A-2D). These findings were consistent with frontal fibrosing alopecia.

In addition, patchy, moth-eaten appearing alopecia was observed on the occipital scalp. Also, there was hair loss of the bilateral eyebrows. However, there were no facial papules and there were no other body areas of alopecia. There were no cutaneous or mucosal lesions of lichen planus.

A comprehensive laboratory assessment was performed. Her vitamin D,25-OH was noted to be decreased at 13 ng/mL (normal, 30-100 ng/mL). In particular, her antinuclear antibody screen was negative, thyroxine normal, and rapid plasma reagin non-reactive. All other lab tests were normal or negative; these included androstenedione, complete blood count, complete metabolic panel, dehydroepiandrosterone sulfate, ferritin, iron, prolactin, testosterone, total iron-binding capacity, and 17-hydroxyprogesterone.

**Figure 3:**
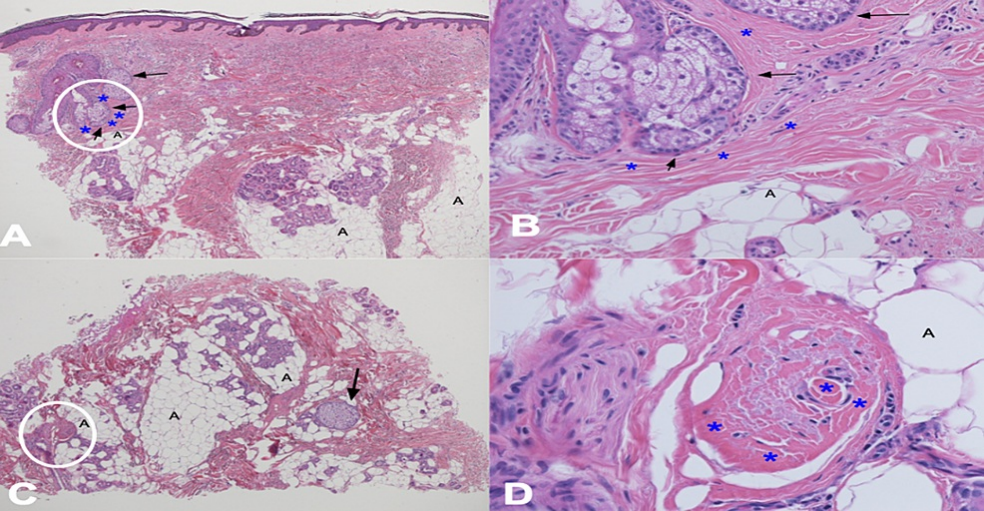
Pathology changes of frontal fibrosing alopecia from the frontal scalp biopsy Distant (A, C) and closer (B, D) views of vertical (A, B) and horizontal (C, D) sections performed on biopsy of the frontal scalp showed areas of the fibrotic dermis (blue asterisks) around sebaceous hair glands (black arrows) associated with hair follicles and areas of adipose tissue (black A). The white ovals (A, C) correspond to the location of the closer views (B, D). The pathologic findings demonstrate a late-stage scarring alopecia; these findings are compatible with late-stage frontal fibrosing alopecia (hematoxylin and eosin: A, x2; B, x20; C, x2; D, x40).

**Figure 4:**
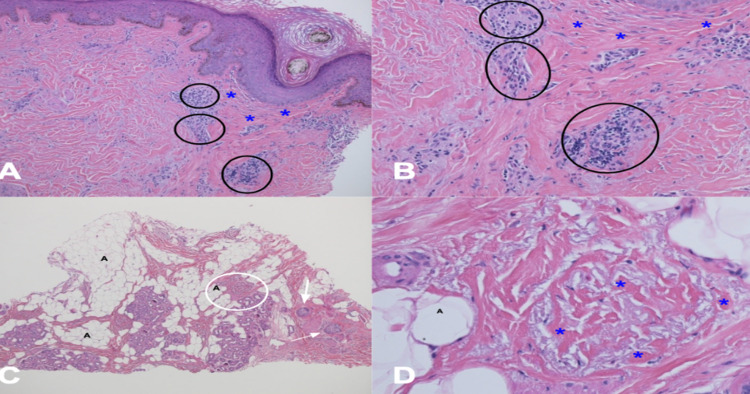
Biopsy from the scalp crown shows frontal fibrosing alopecia changes Distant (A, C) and closer (B, D) views of vertical (A, B) and horizontal (C, D) sections performed on biopsy of the scalp crown show a perifollicular inflammatory cell infiltrate of lymphocytes and histiocytes (black ovals) and areas of the fibrotic dermis (blue asterisks) around sebaceous hair glands (white arrows) associated with hair follicles and areas of adipose tissue (black As). The white oval (C) corresponds to the location of the closer view (D). The pathologic findings demonstrate a late-stage scarring alopecia; these findings are compatible with late-stage frontal fibrosing alopecia (hematoxylin and eosin: A, x2; B, x20; C, x2; D, x40).

Scalp biopsies, for vertical and horizontal processing, were performed from the frontal area of hair loss and the occipital, moth-eaten area of alopecia. Both showed similar features of a late-stage scarring alopecia with extensive loss of follicles with a focal sparse perifollicular inflammatory cell infiltrate of lymphocytes and histiocytes. In addition, there is perifollicular fibroplasia. The periodic acid-Schiff stain was negative for hyphae (Figures 3A-3D, 4A-4D).

Correlation of the clinical presentation, laboratory studies, and pathology findings established diagnosis of frontal fibrosing alopecia with characteristic features (frontal) and atypical pattern of hair loss (occipital); the latter mimicked alopecia syphilitica. She was treated with a daily vitamin D supplement to correct the deficit.

Treatment options for her alopecia were discussed, including oral hydroxychloroquine; she declined this treatment. Therefore, the initial treatment for her hair loss included bilateral intralesional triamcinolone (three milligrams per milliliter, with one milliliter given monthly in each temporal area), minoxidil 5% solution twice daily to the frontal areas of alopecia (except in the areas of intralesional triamcinolone), and doxycycline monohydrate 100 milligrams twice daily.

**Figure 5:**
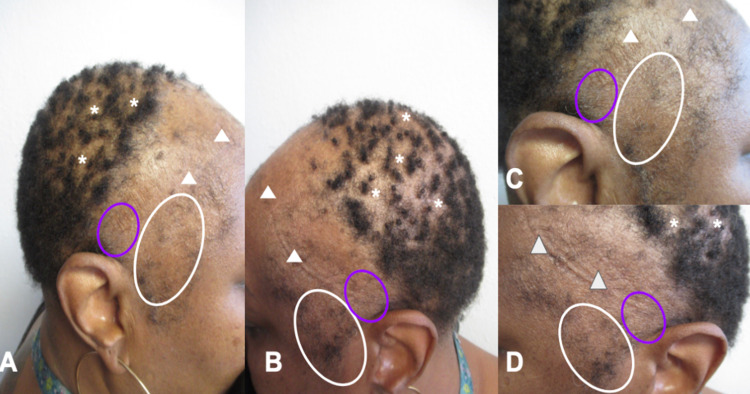
Minimal resolution of frontal fibrosing alopecia Distant (A, B) and closer (C, D) views of the right side (A, C) and left side (B, D) of the scalp two months after treatment. No hair growth occurred at the monthly intralesional triamcinolone injection sites (purple ovals). Focal vellus hair growth was achieved on the right and lateral forehead (white ovals) at the twice-daily minoxidil 5% solution treatment locations. Residual alopecia was observed on the forehead (white triangles) and scalp crown (white asterisks).

The patient returned for monthly intralesional triamcinolone. After two months of treatment, there was some vellus hair growth. However, the hair growth was not at the sites of injection at the temples but inferior on her forehead where she had been applying the minoxidil solution (Figures 5A-5D). She decided to discontinue all therapies and return to wearing a wig.

## Discussion

Frontal fibrosing alopecia typically has been observed in postmenopausal women. However, in a large retrospective study, it was also observed in premenopausal women. It has rarely been observed in men and sometimes children [5].

In the early phases of frontal fibrosing alopecia, some patients are symptomatic. Symptoms include burning and itching. In later phases, patients are usually asymptomatic [6].

There are numerous diseases that have been associated with frontal fibrosing alopecia. The two most common conditions associated with frontal fibrosing alopecia are hypothyroidism and androgenetic alopecia. In addition to thyroid disease, other autoimmune conditions that have been observed are systemic lupus erythematosus and alopecia areata. Other cutaneous conditions that have been seen with frontal fibrosing alopecia are allergic contact dermatitis to fragrance and rosacea [1,2,7].

In addition to frontotemporal hair loss, noninflammatory skin-colored facial papules on the forehead, erythema (perifollicular and diffuse), follicular red dots on the glabella, and frontotemporal depression have been observed. Also, hypopigmentation in the areas affected by frontal fibrosing alopecia hair loss has been noted; this can be more readily appreciated during a Wood light examination. However, in patients with darker skin types, hyperpigmentation has been observed in areas affected by frontal fibrosing alopecia hair loss; this has been postulated to possibly represent current lichen planus pigmentosus [2,7].

Pathologic changes of frontal fibrosing alopecia depend on the duration of the condition. In the acute phase of frontal fibrosing alopecia, a follicular triad has been observed. This includes lymphocytic infiltration around the follicle, perifollicular fibrosis, and loss of the follicle. In later stages of frontal fibrosing alopecia, the features are identical to those of lichen planopilaris and show end-stage scarring alopecia. Therefore, a scalp biopsy for vertical and horizontal sectioning may be helpful in establishing the suspected diagnosis [1,2,7].

The pathogenesis of frontal fibrosing alopecia remains unknown. However, several possible etiologies have been suggested for the development of frontal fibrosing alopecia. It may be multifactorial with autoimmune, environmental, genetic, hormonal and/or inflammatory factors [2].

Criteria have been created for diagnosing frontal fibrosing alopecia (Table 1) [2,8]. The diagnosis is established with either two major criteria or one major criterion and two minor criteria [2]. Recently criteria for frontal fibrosing alopecia in an androgenetic alopecia distribution have been proposed [8].

**Table 1:** Diagnostic criteria for frontal fibrosing alopecia ^a^The diagnosis of frontal fibrosing alopecia requires two major criteria or one major criterion and two minor criteria [2]. ^b^Diagnostic criteria for frontal fibrosing alopecia in a pattern (androgenetic alopecia) distribution has recently been proposed [8].

Criteria^a,b^
Major criteria
1. Cicatricial alopecia of the frontal, temporal, or frontotemporal scalp without the presence of follicular keratotic papules on the body.
2. Diffuse alopecia of the bilateral eyebrows.
Minor criteria
1. Compatible trichoscopic features (perifollicular erythema with or without follicular hyperkeratosis, and lonely hair sign).
2. Histopathologic features of frontal fibrosing alopecia and lichen planopilaris.
3. Hair loss of perifollicular erythema at additional frontal fibrosing alopecia areas (body hair, facial hair, occipital area, or sideburns).
4. Non-inflammatory facial papules.
5. Prior or concurrent symptoms of pain or pruritis at involved sites.

There is not a consistent successful treatment for frontal fibrosing alopecia. Topical management often includes calcineurin inhibitors and corticosteroids; the latter is also frequently used intralesionally. Since concurrent androgenetic alopecia may be present, similar to our patient, topical minoxidil may also be helpful. Oral therapy has included hydroxychloroquine, tetracyclines, or both. In addition, there are several systemic agents that have been used such as finasteride, isotretinoin, methotrexate, mycophenolate mofetil, naltrexone, pioglitazone, prednisone, and tofacitinib [2,9].

There is frequently limited success to the available treatments for frontal fibrosing alopecia; indeed, both the patient and the clinician need to anticipate disappointments or limited results in the response to treatment of this entity. In addition, it can take months to years to respond to attempted therapeutic interventions. Our patient decided to discontinue all therapies after two months; this is likely to be too short a duration of treatment to evaluate the limited to absent results of the therapeutic interventions she received.

The hair loss pattern in frontal fibrosing alopecia is traditionally frontal and temporal. It can be localized or diffuse. It may be asymptomatic or symptomatic. However, it may also include hair loss at other body sites, including the eyebrows (Table 2) [2,4].

**Table 2:** Typical patterns of frontal fibrosing alopecia ^a^Trichoscopy compatible with frontal fibrosing alopecia will show perifollicular erythema and scaling, loss of follicular openings, and presence of lonely hairs.

Pattern	Clinical description
Diffuse	Hair loss affecting the frontal hairline in a zigzag band-like pattern with additional loss of hair density behind the hairline (50 percent or more) with a compatible trichoscopy^a^
Linear	Hair loss affecting the frontal hairline in a uniform band without loss of hair density behind the hairline

Several atypical patterns of hair loss have been observed in patients with frontal fibrosing alopecia (Table 3) [2-4,10]. These include the androgenetic alopecia-like pattern, cockade-like pattern, ophiasis-like pattern, and upsilon pattern. In addition, several critical signs have been observed in patients with frontal fibrosing alopecia: the doll hairline sign, lonely hair sign, and pseudo-fringe sign. Our patient had an unusual pattern of hair loss mimicking that which is observed in patients with alopecia syphilitica; however, her rapid plasma reagin was negative, and her biopsy showed scarring alopecia consistent with frontal fibrosing alopecia [2,3,10,11].

**Table 3:** Unusual patterns and signs of frontal fibrosing alopecia ^a^Androgenetic alopecia is a separate disorder but can concurrently occur with frontal fibrosing alopecia [2,3]. ^b^The pseudo-fringe sign is considered to be one of the typical patterns of frontal fibrosing alopecia by some investigators [2,10].

Pattern or sign	Clinical description	References
Alopecia syphilitica-like pattern	Moth-eaten pattern of hair loss in the occipital region secondary to syphilis.	[2-4]
Androgenetic-like pattern^a^	Hair loss at the frontotemporal hairline in a symmetric pattern and an unaffected paramedian frontal hairline, mimicking male pattern androgenetic alopecia.	[2,3]
Clown alopecia pattern	Complete loss of hair in the frontoparietal area.	[2]
Cockade-like pattern	Unaffected band of hair at the temporal hairline with bilateral oval patches of alopecia in the temporal area.	[2,3]
Doll hairline sign	Absence of intermediate and vellus hairs at the primitive hairline.	[2]
Lonely hair sign	Presence of one or a few isolated terminal hairs at the primitive hairline.	[2]
Ophiasis-like pattern	Continuous hair loss from the frontal to occipital regions along the hairline.	[2,3]
Pseudo-fringe sign^b^	Frontal fibrosing alopecia without hair loss at the frontal or temporal hairline.	[2,10]
Upsilon pattern	A band of alopecia along the frontotemporal scalp extending into two symmetrical triangles onto the parietal scalp.	[2]

Alopecia syphilitica is a pattern of non-scarring hair loss that has been observed in some patients with secondary syphilis. Clinically, its appearance has been described as moth-eaten alopecia. In patients with straight hair, the residual hair strands may partially cover the spots of hair loss; however, in patients with the curly hair, the patches of alopecia may appear more distinct. In addition to hair loss on the scalp, alopecia syphilitica can also cause hair loss at the eyebrows, eyelashes, and other body sites [4,12,13].

Alopecia syphilitica is associated with serological evidence, such as a positive rapid plasma reagin, of syphilis. In addition to frontal fibrosing alopecia, the differential diagnosis of alopecia syphilitica includes alopecia areata, alopecia neoplastica, tinea capitis, and trichotillomania. The treatment of alopecia syphilitica in non-complicated secondary syphilis in an immunocompetent patient usually includes an injection of intramuscular penicillin. In patients allergic to penicillin, doxycycline has been used [12].

The patient in this report had two patterns of frontal fibrosing alopecia hair loss. The frontotemporal pattern was consistent with that typically observed in frontal fibrosing alopecia. The occipital pattern masqueraded as alopecia syphilitica; however, her rapid plasma reagin was negative and the biopsy demonstrated scarring alopecia, consistent with frontal fibrosing alopecia. Her hair loss did not improve with conservative treatment and she elected to discontinue therapy and return to wearing a wig.

In summary, frontal fibrosing alopecia is characterized by typical and atypical patterns of hair loss. The typical patterns are diffuse and linear. In addition to an androgenetic alopecia-like pattern, cockade-like pattern, ophiasis-like pattern, and upsilon pattern, alopecia syphilitica-like pattern can now be added to the atypical hair loss patterns observed in patients with frontal fibrosing alopecia.

## Conclusions

Frontal fibrosing alopecia is a primary scarring alopecia that is postulated to be a clinical variant of lichen planopilaris. Alopecia syphilitica is a less common manifestation of secondary syphilis that presents with alopecia in a moth-eaten pattern. Frontal fibrosing alopecia has a classic presentation characterized by regression of the frontotemporal hairline. However, albeit less frequently, hair loss in frontal fibrosing alopecia can be atypical. The woman in this report had an atypical pattern of frontal fibrosing alopecia hair loss that masqueraded as alopecia syphilitica. However, her rapid plasma reagin was negative. In addition, scalp biopsies from not only the frontal area of classic appearing hair loss but also the occipital area with an atypical pattern of hair loss both demonstrated changes of frontal fibrosing alopecia. Therefore, alopecia syphilitca-like patterns of hair loss can be added to the atypical patterns of hair loss associated with frontal fibrosing alopecia.
